# Supplementary material for: Engineering the auxin-inducible degron system for tunable in vivo control of organismal physiology
Source: Nat Commun. 2025 Dec 12;16:10848. doi: 10.1038/s41467-025-66347-x (PMC12700905; doi:10.1038/s41467-025-66347-x)
Supplement: Supplementary file 1 — Supplementary Information [file 41467_2025_66347_MOESM1_ESM.pdf]

Supplementary Information

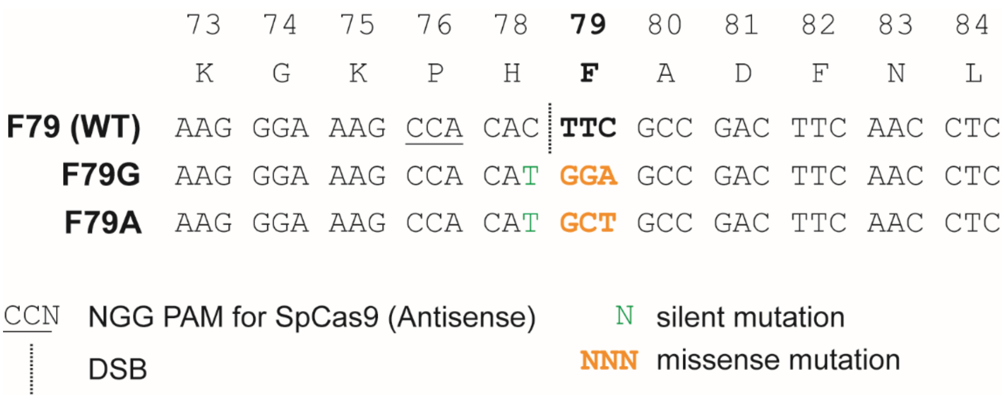

Supplementary Figure 1: **Transgene design —** Conversion of the wild-type *At*TIR1 F79 residue to F79G and F79A using CRISPR–Cas9. *Sp*Cas9, directed by a guide RNA (5'–AGGTTGAAGTCGGCGAAGTG–3'), produces a double-strand break (DSB) immediately upstream of the codon coding for the phenylalanine residue at amino acid position 79. Single-stranded oligodeoxynucleotide (ssODN) donors were used to introduce the corresponding mutations (yellow) that convert phenylalanine to glycine or alanine via homology-directed repair (HDR). Moreover, an additional silent mutation (green) was introduced in amino acid position 78 (c.238C>T) to facilitate genotyping using amplification-refractory mutation system PCR (ARMS-PCR).

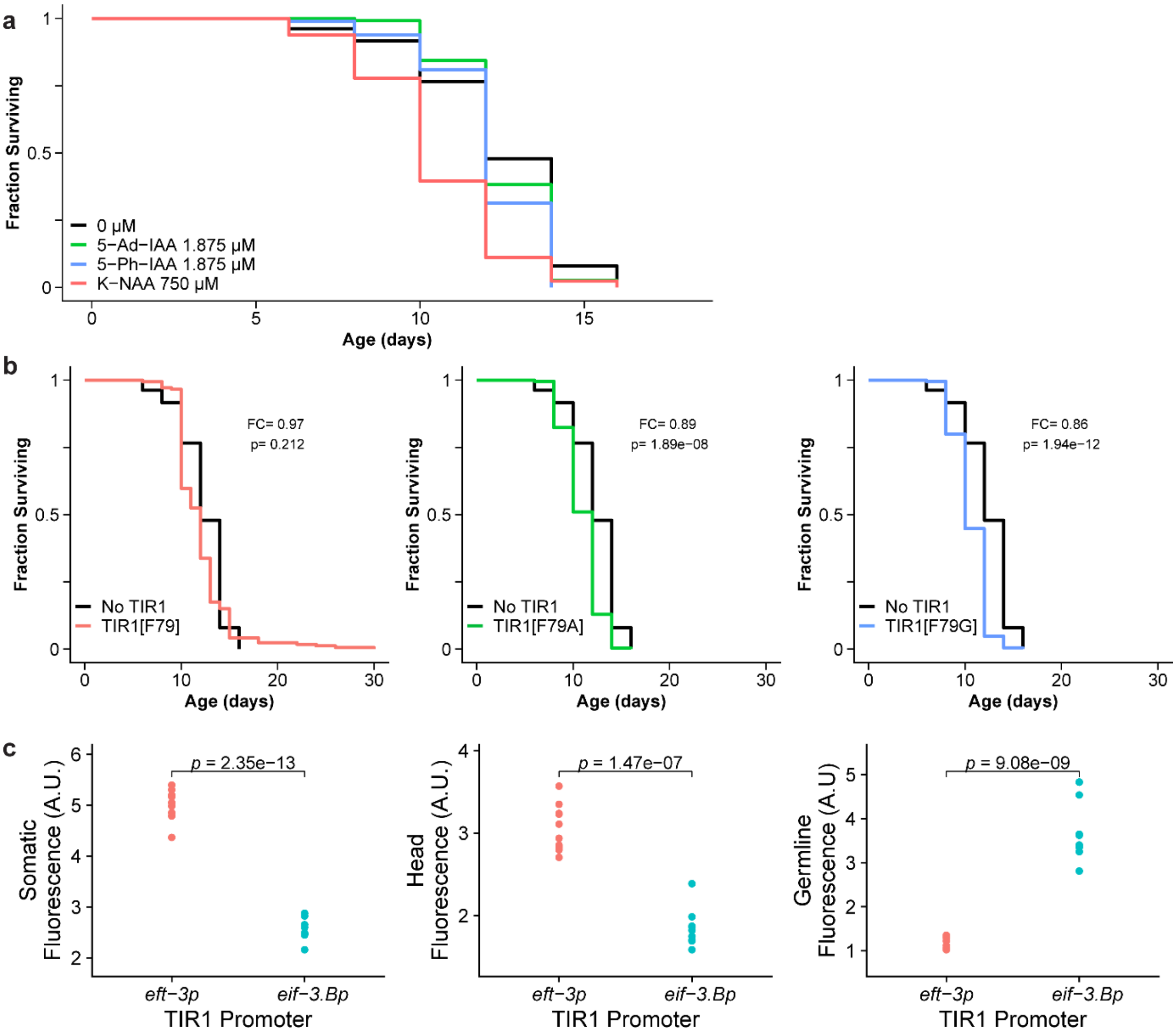

Supplementary Figure 2: **The quantitative activities of TIR1 variants— a.** The effect of 750 µM K-NAA (*red,* N = 81 individuals)*,* 1.875 µM 5-Ad-IAA (*green*, N = 115 individuals), and 1.875 µM 5-Ph-IAA (*blue*, N = 99 individuals) on wild-type *C. elegans* lifespan. **b.** The effect of expression of TIR1[F79], TIR1[F79A], and TIR1[F79G] transgenes in the presence of DAF-2::1xAID on lifespan in the absence of activating compound, annotated by the fold-change effect on lifespan and *p*-value (Accelerated Failure time Regression model, *Statistical Methods*) (N = 228, 240, and 213 individuals, respectively from three independent experiments). **c.** Quantification of the *eft-3p*::TIR1::mRuby and *eif-3.Bp*::TIR1::mRuby transgenes shown in Fig. 2b. Fluorescence was quantified in images masked to contain only the somatic tissues of each of 10 individuals *(left)*, only the head *(center)*, and only the germline *(right)* from a single experiment.

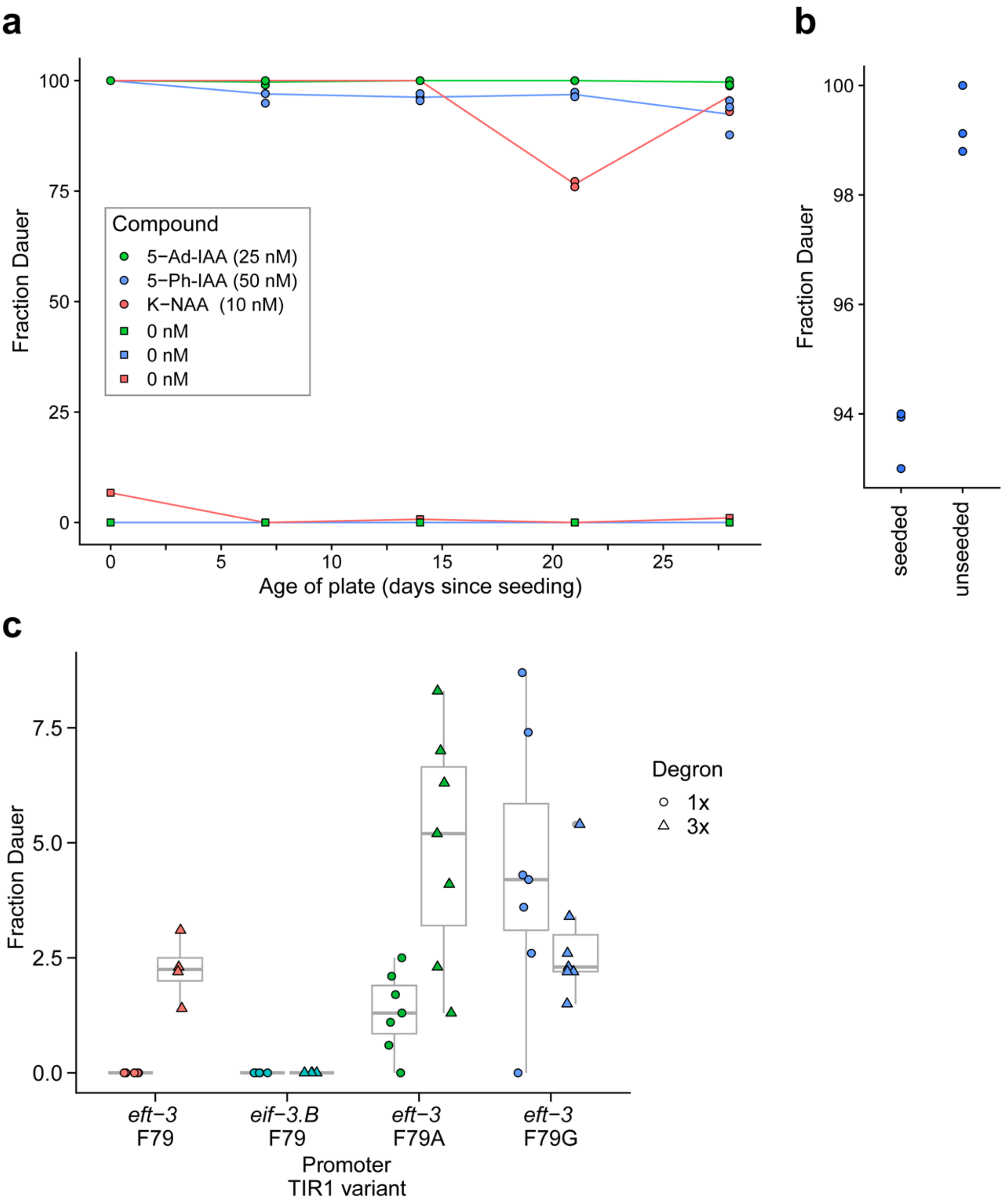

Supplementary Figure 3: Compound degradation and TIR1 basal activity — a. Plates containing each compound, 5-Ad-IAA (*green*), 5-Ph-IAA (*blue*), and K-NAA (*red*) were seeded with OP50 bacteria and stored at room temperature. As the plates aged, fresh *daf-2::1xAID; TIR1(v)* nematodes were prepared, with *v* being the sensitive variant for each compound, F79 (*red*), F79G (*blue*) and F79A (*green*) (N = 6609 individuals from a single experiment). In this way, any decrease in effective compound activity would be revealed as a decrease in the fraction of individuals entering dauer due to DAF-2 degradation. **b.** The effect of seeding on K-NAA plates tested after 28 days. **c.** Auxin-independent effects in different promoter–TIR1 variant combinations in the dauer assay. Even in the absence of auxin, some worms proceed into dauer entry, suggesting the occurrence of DAF-2 basal degradation that results in dauer formation. The weakly expressing *eif-3.Bp::TIR1* does not result in dauer formation, regardless of degron tag number. In contrast, the strongly expressing *eft-3p::TIR1* results in dauer formation in all TIR1 variants when there are three degrons, and when there is a single degron in both F79A and F79G, but not in F79. The data is obtained from a single trial with seven replicates each.

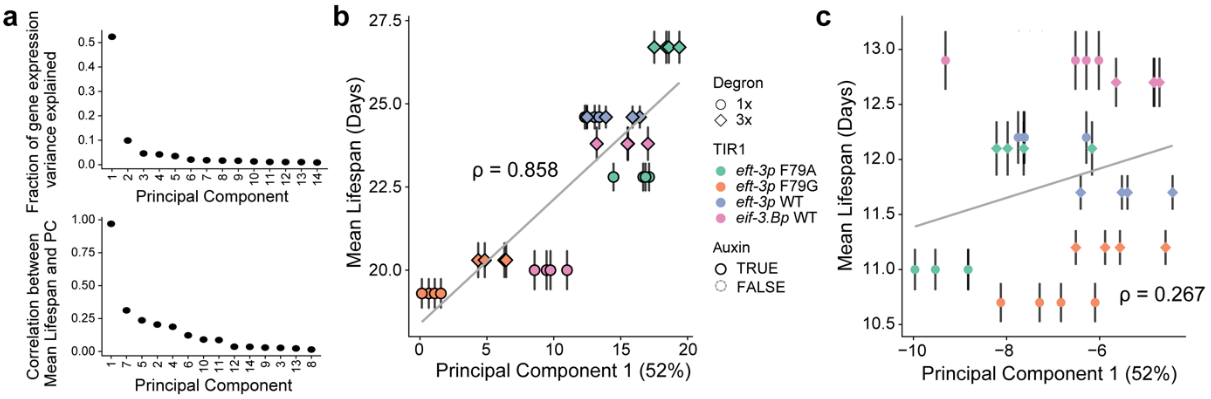

Supplementary Figure 4: Comparing the "on-target" effects of AID system variants— Principal component analysis (PCA) was performed on the transcriptomes of day 1 young adults to determine principal components (PCs) which account for transcriptomic variation. **a.** The fraction of gene expression variance explained by the first 14 PCs across all TIR1 variants (*upper*) or the correlation between each PC and mean lifespan (*lower*). **b.** The correlation between PC1 and mean lifespan considering only auxin-dependent effects or **c.** auxin-independent effects.

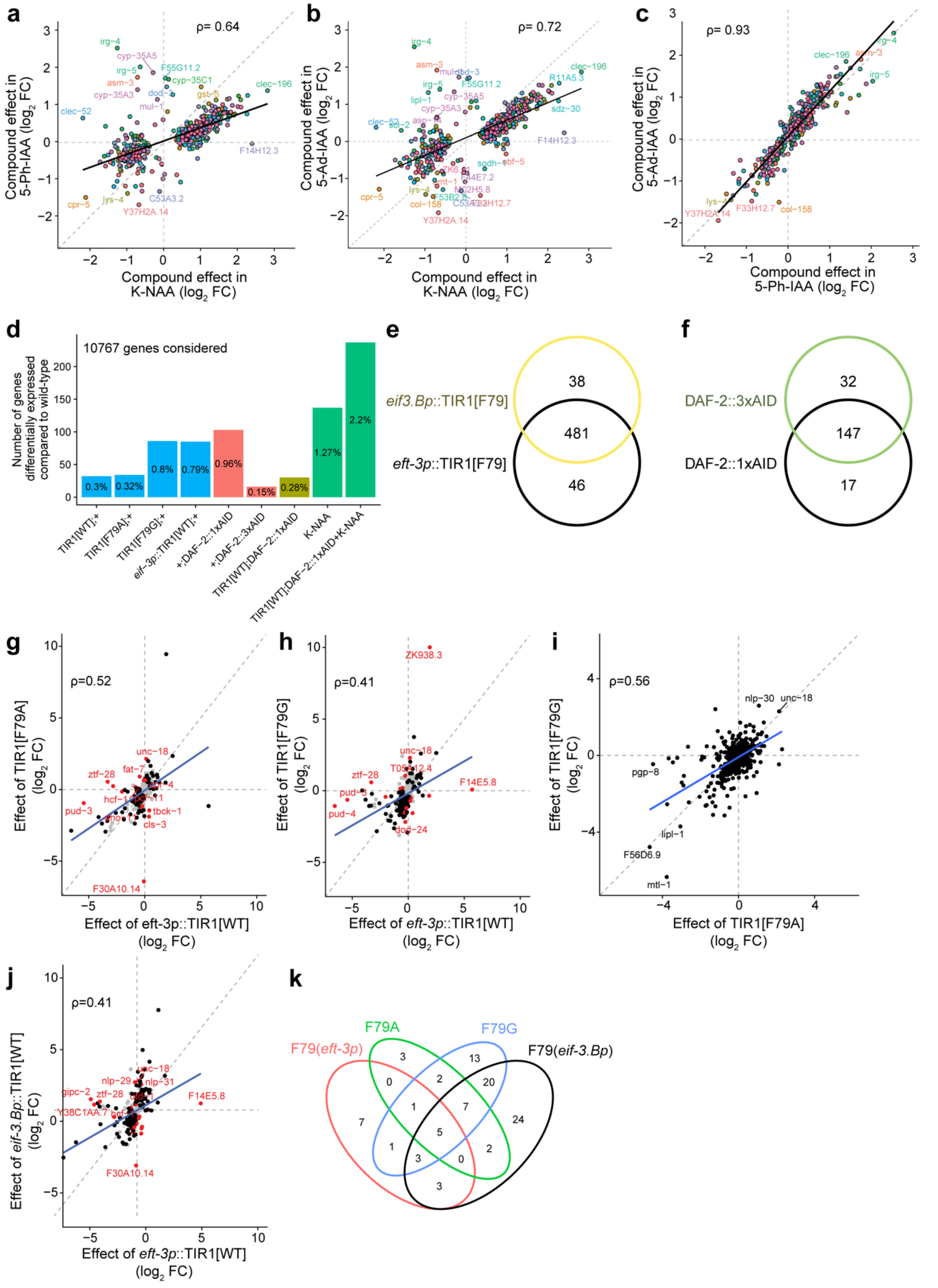

Supplementary Figure 5: Comparing the "off-target" effects and basal activity of AID system variants—a. Genes differentially regulated by K-NAA in wild-type individuals, compared to the effect of 5-Ph-IAA or **b.** the effect of 5-Ad-IAA. Each point is a gene. **c.** The effects of 5-Ph-IAA compared to 5-Ad-IAA. **d.** The number of differentially regulated genes in

the absence of any activating compounds, observed in day 1 adults expressing only a TIR1 variant (*blue*), only the 1x or 3x *daf-2*::AID tag (*red*), or in the double TIR1[F79]; *daf-2*::1xAID (*yellow*), or by K-NAA alone or in the double activated by K-NAA (*green*). **e.** The effect on gene-expression of the *daf-2::1xAID* alone (without any other AID system component) (*x-axis*) compared to the effects of *daf-2::3xAID* alone (*y-axis*). **f.** The Venn diagram of the number of genes significantly differentially expressed by either the *daf-2*::1xAID or *daf-2*::3xAID in the absence of any other AID system component. **g.** The effect on gene expression of the *eft-3p*::TIR1[F79] transgene alone (without any other AID system components) (*x-axis*), compared to the effect of *eft-3p*::TIR1[F79A] alone (*y-axis*). Genes showing statistically significant differences in their response to F79 and F79A are highlighted in red. **h.** the same analysis as *g*, but comparing the effects of *eft-3p*::TIR1[F79] (*x-axis*) to *eft-3p*::TIR1[F79G] (*y-axis*). **i.** the same analysis as *g*, but comparing the effects of *eft-3p*::TIR1[F79A] (*x-axis*) to *eif-3.Bp*::TIR1[F79G] (*y-axis*). **j.** the same analysis as *g*, but comparing the effects of *eft-3p*::TIR1[F79] (*x-axis*) to *eif-3.Bp*::TIR1[F79] (*y-axis*). **k.** The Venn diagram of the effects of each TIR1 variant alone (without any other AID system components) on gene expression, counting significantly differentially expressed genes.

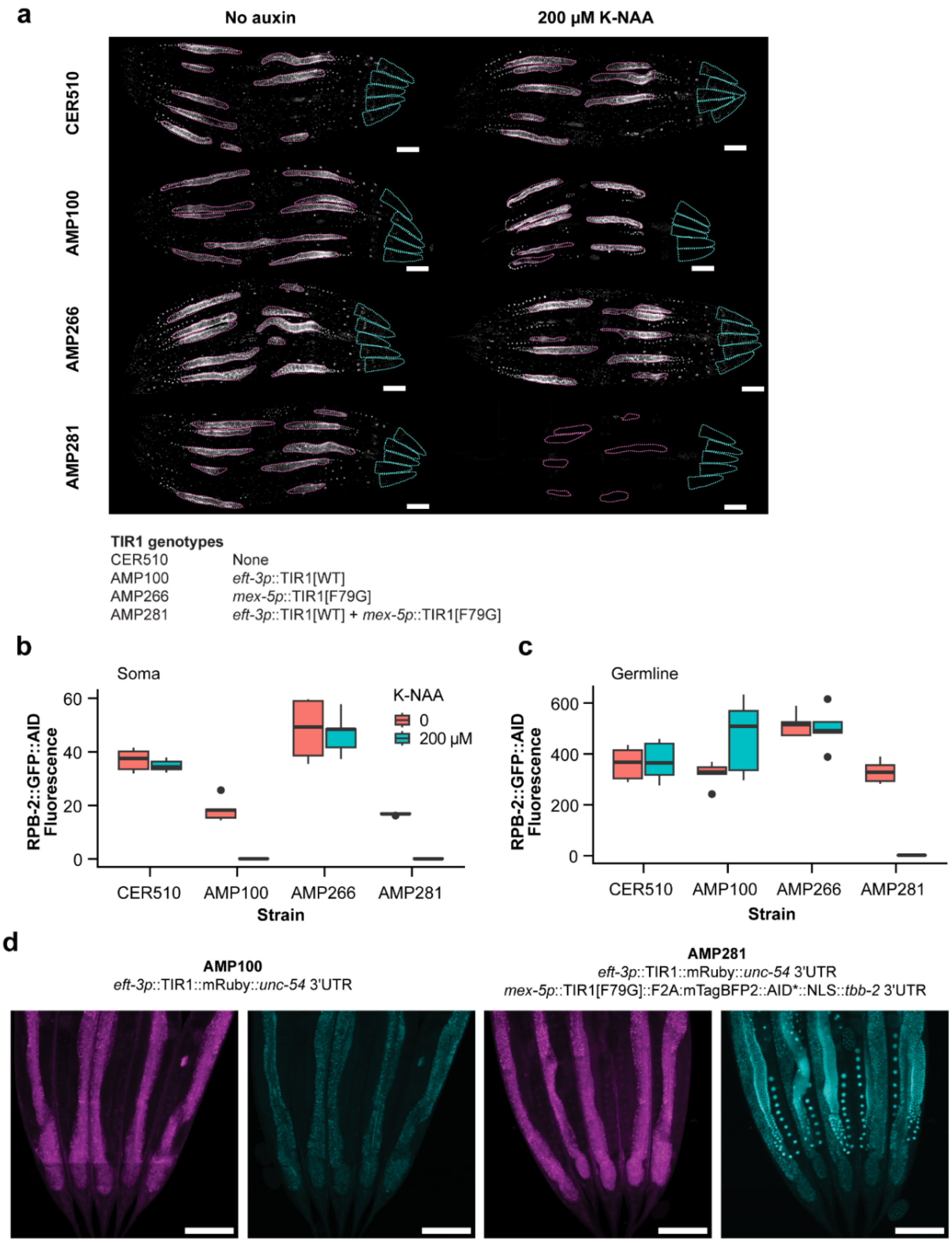

Supplementary Figure 6: **Trans-activation of *eft-3p*::TIR1::mRuby::*unc-54* 3'UTR in the germline in the presence of *mex-5p*::TIR1[F79G]::*tbb-2* 3’UTR in the dual-channel strain AMP281**—**a.** Representative images of worms with RPB-2::GFP::AID combined with different TIR1 genotypes without auxin or on 200 µM K-NAA. **b.** Quantification of RPB-2::GFP signal in the soma or **c.** in the germline of worms shown in (a). The signal is quantified in absolute photon counts from five worms per condition from a single biological replicate. **d.** Comparison of *eft-3p*::TIR1:: mRuby::*unc-54* 3'UTR signal in the germline in the absence (AMP100) or presence (AMP281) of *mex-5p*::TIR1[F79G]::F2A:: mTagBFP2::AID*::NLS:: *tbb-2* 3’UTR. TIR1::mRuby and TIR1[F79G]:: mTagBFP2 are imaged in the red and blue channels, respectively. Scale bars = 100 µm.

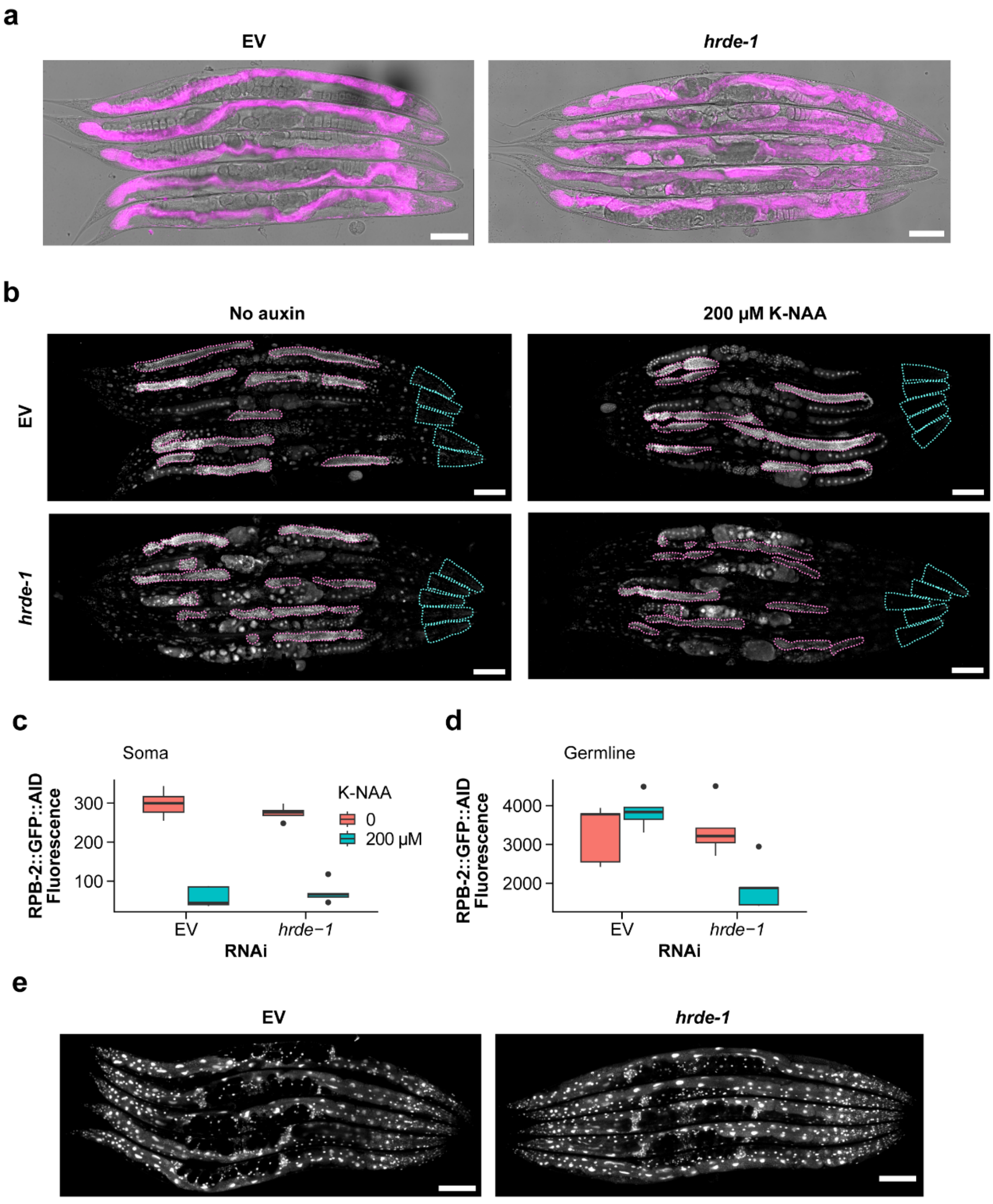

Supplementary Figure 7: De-silencing of TIR1 in the germline upon exposure to *hrde-1* RNAi—a. De-silencing of *eft-3p*::TIR1::mRuby::*unc-54* 3'UTR in the germline of worms exposed to five generations of *hrde-1* RNAi. **b.** Depletion of degron-tagged RPB-2::GFP signal in worms with *eft-3p*::TIR1::mRuby::*unc-54* 3'UTR when exposed to five generations of *hrde-1* RNAi and three hours of 200 µM K-NAA. Quantification of RPB-2::GFP signal in the **c.** soma (head) or **d.** germline of worms shown in (b). The signal is quantified in absolute photon counts from five worms per condition from a single biological replicate. **e.** De-silencing of *eft-3p*::TIR1::SL2::NLS::mTagBFP2::*tbb-2* 3'UTR in the germline upon exposure to *hrde-1* RNAi for eight generations. Scale bars = 100 µm.

Supplementary Table 1: List of differentially expressed genes in wild-type QZ0 worms upon exposure to auxin analogs (full differential expression data included as "Supplementary Data 1 – compound effects.csv")

K-NAA			5-Ad-IAA	5-Ph-IAA
B0281.5	Y58A7A.5	*jmjd-3.2*	B0281.5	F55G11.2
C06E2.5	Y62H9A.15	*lbp-7*	C08F1.6	K02C4.8
C07D10.5	Y71F9AL.7	*lec-9*	C17E4.2	W07E6.5
C08F1.6	ZK185.9	*lfor-1*	F33H12.7	Y37H2A.14
C10C5.4	ZK550.2	*lido-8*	F53B2.8	*adh-1*
C10G8.4	*abts-4*	*mnp-1*	F55G11.2	*asah-1*
C14H10.2	*acdh-1*	*nhr-2*	K02B12.2	*asm-3*
C17C3.5	*ags-3*	*nlp-31*	M02H5.8	*clec-47*
C17E4.2	*aptf-4*	*noah-1*	R11A5.3	*cyp-35A3*
C17E4.20	*arrd-1*	*oac-32*	T25E12.6	*cyp-35A5*
C31H5.4	*arrd-2*	*phdh-1*	Y37H2A.14	*cyp-35C1*
C33G3.4	*asp-5*	*pmp-1*	Y46G5A.7	*dod-3*
C34H4.1	*atz-1*	*scl-2*	*acdh-1*	*gst-5*
C35E7.5	*btb-11*	*scl-24*	*amt-1*	*hmit-1.1*
C39B5.2	*btb-6*	*sdz-14*	*asah-1*	*irg-4*
C50B6.7	*btb-8*	*sdz-21*	*asm-3*	*irg-5*
D1086.7	*cav-1*	*sdz-28*	*btb-6*	*lfor-1*
E02C12.8	*cec-8*	*sdz-30*	*btb-8*	*lys-4*
E04F6.15	*ceh-39*	*sea-1*	*clec-196*	*nlp-31*
F08F1.4	*cest-13*	*sepa-1*	*clec-47*	*pgph-3*
F09F7.6	*cht-1*	*skr-10*	*col-158*	
F14B6.3	*clc-23*	*skr-13*	*cyp-35A5*	
F14H12.3	*clec-196*	*skr-14*	*cyp-35C1*	
F15B10.3	*clec-265*	*skr-15*	*dod-3*	
F15D4.5	*clec-52*	*skr-7*	*epg-2*	
F17C11.6	*clec-62*	*skr-8*	*fbxb-26*	
F22E5.20	*clec-67*	*skr-9*	*fbxc-36*	
F31E8.4	*cpr-4*	*spp-25*	*fmo-1*	
F33E2.5	*cpr-5*	*sptl-2*	*hmg-11*	
F39F10.3	*cpr-9*	*sup-36*	*inx-2*	
F45B8.6	*cyd-1*	*tatn-1*	*irg-4*	
F48C1.9	*cyn-6*	*tbx-11*	*irg-5*	
F48E3.6	*dhs-9*	*tipn-1*	*lipl-1*	
F59A6.12	*die-1*	*ttr-20*	*lys-4*	
H06H21.8	*drd-5*	*ttr-21*	*mul-1*	
H37A05.4	*dsl-2*	*ttr-50*	*pgph-3*	
K02B12.2	*duxl-1*	*ugt-26*	*pmp-1*	
K04G2.10	*epg-2*	*ule-1*	*sdz-28*	
K10D11.5	*fbxb-10*	*ule-2*	*skr-10*	
M02D8.3	*fbxb-15*	*vet-1*	*skr-7*	
R11A5.3	*fbxb-26*	*vet-2*	*skr-9*	
T05D4.2	*fbxb-91*	*vet-6*	*tatn-1*	
T09B4.5	*fbxc-21*		*tipn-1*	
T25E12.6	*fbxc-36*		*vet-6*	
T28F3.8	*fbxc-51*			
W04A8.4	*fmo-1*			
Y106G6D.1	*gem-4*			
Y106G6D.2	*gmap-1*			
Y15E3A.5	*gpr-1*			
Y27F2A.8	*gst-5*			
Y32F6A.4	*hacd-1*			
Y38H6C.15	*hil-2*			
Y45G5AM.5	*hil-3*			
Y46G5A.20	*his-24*			
Y46G5A.7	*hmg-11*			
Y46H3C.5	*hmit-1.1*			
Y46H3C.7	*hsp-16.41*			
Y51A2D.13	*inx-2*			

Supplementary Table 2: List of differentially expressed genes in strains with different transgenes at the TIR1 and *daf-2* loci in the absence of K-NAA, 5-Ph-IAA, and 5-Ad-IAA (full differential expression data included in the file "Supplementary Data 2 - degron component effects.csv")

TIR1[F79] ; +	TIR1[F79A] ; +	TIR1[F79G] ; +	eif-3p:: TIR1[F79] ; +	+ ; DAF-2:: 1xAID	+ ; DAF-2:: 3xAID
C55C3.3	F30A10.14	C14C6.2	C06B3.6	B0205.13	C55C3.3
F09C8.1	**F42A9.6***	C14C6.5	C14C6.2	C06B3.6	F19B2.5
F14E5.8	**H19N07.3***	C53A3.2	C53A3.2	C14C6.2	**H19N07.3****
F19B2.5	Y48E1B.8	F30A10.14	F09E10.1	C14C6.5	**T03D3.5****
**F42A9.6***	ZK938.3	F35F10.5	F22B7.9	C53A3.2	**Y38C1AA.7****
**H19N07.3***	asp-12	**F42A9.6***	F30A10.14	F09C8.1	Y94H6A.10
R07E3.1	atp-6	F49C12.14	F41C3.2	F15E6.3	col-129
T28C12.4	cls-3	F52E1.14	**F42A9.6***	F41C3.2	**col-139****
Y102A5C.6	col-135	F55B11.4	F56A8.8	F49C12.14	**col-81****
Y38C1AA.7	ctb-1	**H19N07.3***	**H19N07.3***	**H19N07.3****	dyf-3
Y94H6A.10	ctc-1	K09C6.9	R11A5.3	H34I24.2	**msd-4****
acs-2	ctc-3	T01D3.6	T01D3.6	M28.10	msp-40
asp-8	dod-24	T03D3.5	T03D3.5	**T03D3.5****	**msp-76****
gipc-2	ins-33	T05A12.4	T22B7.7	T05E12.3	msrp-2
gst-10	lys-7	T22B7.7	T28C12.4	T28C12.4	nspd-1
msd-4	nduo-6	W06A11.4	Y102A5C.6	W01F3.2	pcf-11
msp-76	pcf-11	Y102A5C.6	Y119D3B.13	Y119D3B.13	pud-3
pud-3	pho-11	Y119D3B.13	Y25C1A.6	Y34B4A.5	**ssq-1****
pud-4	sepa-1	Y34B4A.5	Y34F4.1	**Y38C1AA.7****	**ssq-2****
sax-2	ssq-1	Y34F4.1	Y41G9A.10	Y41G9A.10	
ssq-1	tbck-1	ZK673.1	ZK938.3	ZK673.1	
ssq-2	**thn-2***	ZK938.3	clec-190	ZK970.7	
**thn-2***	twk-1	aagr-1	clec-7	aagr-1	
ztf-28	unc-18	asah-1	cls-3	asah-1	
		asah-2	cnc-2	asah-2	
		clc-1	cnc-4	asp-17	
		clec-218	col-129	btb-9	
		clec-50	col-139	clec-160	
		clec-7	col-95	clec-7	
		cls-3	dod-23	clec-85	
		col-135	dod-24	cnc-2	
		col-92	dsl-2	cnc-4	
		col-95	fipr-22	cnp-3	
		cpr-1	ges-1	**col-139****	
		cpr-3	grd-10	**col-81****	
		dod-23	hil-3	ctsa-1	
		dod-24	hrg-1	fipr-22	
		fipr-22	ifc-1	gba-4	
		lipl-5	lips-10	ges-1	
		lips-10	lys-7	gst-10	
		lys-7	nlp-29	ifc-1	
		nlp-29	nlp-30	lipl-5	
		nlp-30	nlp-31	lips-10	
		nlp-31	ora-1	lys-7	
		pcf-11	pcf-11	**msd-4****	
		phat-3	pho-11	**msp-76****	
		pho-11	pud-3	nlp-29	
		sax-2	ram-2	nlp-30	
		scl-2	scl-2	nlp-31	
		spp-2	sepa-1	ora-1	
		tcl-2	spp-1	papl-1	
		**thn-2***	spp-2	scl-2	
		unc-119	swt-3	spp-2	
		unc-18	tbck-1	**ssq-1****	
		vit-1	**thn-2***	**ssq-2****	
		vit-3	ttr-44	sta-2	
			ugt-64	swt-3	
			unc-18	tag-10	
			vit-1	thn-2	
			zip-10	ttr-26	
				ttr-33	
				ttr-44	
				tyr-1	
				vhp-1	

*** Genes affected by all four TIR1 variants**

**** Genes affected by both DAF-2::AID tags**

Supplementary Table 3: List of differentially expressed genes in DAF-2::AID; TIR1 strains without exposure to any activating compounds.

eft-3p::TIR1[F79] ; *daf-2*::1xAID		*eft-3p*::TIR1[F79] ; *daf-2*::3xAID		*eif-3.Bp*::TIR1[F79] ; *daf-2*::1xAID		*eif-3.Bp*::TIR1[F79] ; *daf-2*::3xAID
C17C3.5	asp-17	B0035.13	ckr-2	B0272.4	ZK550.2	F14E5.8
F01D5.1	clec-52	C09G5.7	dod-24	C08A9.10	acl-12	F15E6.3
F09C8.1	clec-86	C10G8.4	dsl-3	C31H5.6	acox-1.6	F45D3.4
F14E5.8	drd-5	C50B6.7	fbxb-97	F08F1.4	acox-3	F59C6.16
F35D11.3	fat-5	F19B10.13	linc-6	F14E5.8	asp-13	W10G11.2
F49C12.14	fbxb-97	F26D11.1	lipl-2	F15E6.3	eppl-1	Y73F8A.26
F55F3.2	irg-3	F53A9.8	pud-4	F32D8.12	fat-5	cyp-34A2
M28.10	lys-7	H05C05.1	spp-2	F49C12.14	fat-7	eppl-1
R07E3.1	oac-32	K09D9.1	ttn-1	F59C6.16	fbxb-97	fat-5
T05E12.6	pmp-1	T05E12.6	ttr-33	H06H21.8	gst-10	fbxb-97
T24A6.7	pud-3	W02B8.2	ule-2	R06C1.4	irg-3	irg-3
W02B8.2	pud-4	Y22D7AR.10	ule-4	R07E3.1	lbp-7	mop-25.3
Y17D7C.3	tsp-1	Y38H6C.15	unc-18	T05E12.3	pmp-1	mtl-1
Y34B4A.5	ugt-26	acdh-1		T13F3.6	pud-3	nspe-1
Y37A1B.17	ugt-43			W10G11.2	pud-4	pud-3
acdh-1				W10G11.3	rimb-1	pud-4
				Y62H9A.15		

eft-3p::TIR1[F79G], *daf-2*::1xAID		*eft-3p*::TIR1[F79G] ; *daf-2*::3xAID	*eft-3p*::TIR1[F79A] ; *daf-2*::1xAID	*eft-3p*::TIR1[F79A] ; *daf-2*::3xAID
B0035.13	dao-2	C40H1.7	irg-4	B0238.12
C03G6.5	dod-24	F15E6.3	kqt-2	C03G6.5
C05D2.8	faah-2	F30A10.14	lbp-7	C28C12.4
C08F11.13	fat-7	W02B8.2	lfor-1	C31H5.6
C08G9.2	fmo-1	Y37A1B.17	lys-4	F14H12.3
C09F9.2	frm-9	ZC395.5	mig-6	F15B10.3
C10C5.4	gcy-14	ZK938.3	mtl-1	F41E6.15
C23H3.9	gcy-22	fbxb-97	mtl-2	K09D9.1
C25F9.2	gpr-1	his-64	nlp-31	T05E12.6
C30G12.2	gst-4	nspe-1	nnt-1	T22B7.7
C42D4.1	hil-3	qdpr-1	nspc-13	T24A6.7
E04F6.15	his-64	unc-18	nspe-1	T25G12.6
F07H5.8	hsp-1		pgp-8	T28F3.8
F08F1.4	hsp-60		pqn-60	W02B8.2
F10E9.12	irg-5		pud-3	Y32F6A.4
F14H12.3	kqt-2		pud-4	Y34F4.1
F26E4.5	lbp-7		rpr-1	asp-14
F40F12.7	lipl-2		scl-2	asp-17
F41C3.2	lys-3		skr-10	cpna-2
F53A9.8	mai-1		skr-13	dao-2
F53B2.8	mtl-1		smd-1	dod-24
F59A6.12	nnt-1		spp-2	dod-3
H19N07.3	nspe-1		spp-4	drd-5
K03B8.6	pgp-3		sup-36	fat-7
M04C3.1	pgp-5		sup-46	fbxb-96
MTCE.33	pitr-2		tatn-1	hacd-1
T05E12.6	pmp-1		thn-2	his-64
T22B7.7	pqn-25		timm-17B.2	hsp-43
Y106G6D.8	pud-3		trap-2	irg-5
Y17G9A.4	pud-4		ttr-49	nspe-1
Y32F6A.4	qdpr-1		ttr-50	oac-32
Y32F6B.1	sams-1		tts-1	spp-2
Y54G11A.3	spp-2		ule-2	tag-276
Y57A10A.14	spp-3		unc-18	unc-18
Y62H9A.15	srh-304		vet-6	vet-6
ZC395.5	sulp-8			
ZK550.2	sup-46			
ZK938.3	tag-276			
acdh-1	tatn-1			
acr-19	timm-17B.2			
arr-1	twk-11			
attf-3	ule-2			
che-3	unc-18			
clec-78	vet-6			
cpna-2	ztf-28			
cyp-34A9				

Supplementary Table 4: List of strains used in this study

Strain Name	Genotype	Source
AMP100	*ieSi57 [eft-3p::TIR1::mRuby::unc-54 3'UTR] II; rpb-2(cer135[rpb-2::GFP$^{\Delta piRNA}$::AID::3xFLAG]) III*	Natasha Oswal et al., 2022
AMP145	*ieSi57 [eft-3p::TIR1::mRuby::unc-54 3'UTR + Cbr-unc-119(+)] II; daf-2(ohm13)[daf-2::AID::3xFLAG] III*	This study
AMP158	*ohm10[eft-3p::TIR1[F79A]::mRuby::unc-54 3'UTR + Cbr-unc-119(+)] II*	This study
AMP167	*ieSi57 [eft-3p::TIR1::mRuby::unc-54 3'UTR + Cbr-unc-119(+)] II; daf-2(ohm17)[daf-2::3xAID::3xFLAG] III*	This study
AMP169	*weSi174[eif-3.Bp::TIR1::linker::mCherry$^{\Delta piRNA}$::tbb-2 3'UTR; unc-119(+)] II; daf-2(ohm17)[daf-2::3xAID::3xFLAG] III*	This study
AMP175	*ohm24(unc-119p::TIR1[F79G]::mRuby) IV*	This study
AMP184	*ohm8[eft-3p::TIR1[F79G]::mRuby::unc-54 3'UTR + Cbr-unc-119(+)] II*	This study
AMP205	*weSi174[eif-3.Bp::TIR1::linker::mCherry$^{\Delta piRNA}$::tbb-2 3'UTR; unc-119(+)] II; daf-2(ohm13)[daf-2::AID::3xFLAG] III*	This study
AMP206	*ieSi61 [ges-1p::TIR1::mRuby::unc-54 3'UTR + Cbr-unc-119(+)] II; ohm24(unc-119p::TIR1[F79G]::mRuby) IV; hcf-1(cer159[hcf-1::GFP$^{\Delta piRNA}$::degron::3xFLAG]) IV*	This study
AMP207	*ohm8[eft-3p::TIR1[F79G]::mRuby::unc-54 3'UTR + Cbr-unc-119(+)] II; daf-2(ohm17)[daf-2::3xAID::3xFLAG] III*	This study
AMP208	*ohm10[eft-3p::TIR1[F79A]::mRuby::unc-54 3'UTR + Cbr-unc-119(+)] II; daf-2(ohm17)[daf-2::3xAID::3xFLAG] III*	This study
AMP216	*ohm8[eft-3p::TIR1[F79G]::mRuby::unc-54 3'UTR + Cbr-unc-119(+)] II; daf-2(ohm13)[daf-2::AID::3xFLAG] III*	This study
AMP217	*ohm10[eft-3p::TIR1[F79A]::mRuby::unc-54 3'UTR + Cbr-unc-119(+)] II; daf-2(ohm13)[daf-2::AID::3xFLAG] III*	This study
AMP245	*ohm49[eft-3p::nTIR1::SL2::NLS::mTagBFP2::tbb-2 3'UTR] IV*	This study
AMP266	*ohm52 [mex-5p::TIR1[F79G]::F2A::mTagBFP2::AID*::NLS::tbb-2 3'UTR] I rpb-2(cer135[rpb-2::GFP$^{\Delta piRNA}$::AID::3xFLAG]) III*	This study
AMP267	*ohm53[eft-3p::nTIR1::SL2::NLS::mTagBFP2::eft-3 3'UTR] IV*	This study
AMP281	*ohm52 [mex-5p::TIR1[F79G]::F2A::mTagBFP2::AID*::NLS::tbb-2 3'UTR] I; ieSi57 [eft-3p::TIR1::mRuby::unc-54 3'UTR + Cbr-unc-119(+)] II; rpb-2(cer135[rpb-2:: GFP$^{\Delta piRNA}$::AID::3xFLAG]) III*	This study
AMP284	*rpb-2(cer135[rpb-2::GFP$^{\Delta piRNA}$::AID::3xFLAG]) III; ohm53[eft-3p::nTIR1::SL2::NLS::mTagBFP2::eft-3 3'UTR] IV*	This study
CA1200	*ieSi57 [eft-3p::TIR1::mRuby::unc-54 3'UTR + Cbr-unc-119(+)] II; unc-119(ed3) III*	Abby Dernburg (CGC)
CA1209	*ieSi61[ges-1p::TIR1::mRuby::unc-54 3'UTR + Cbr-unc-119(+)] II; unc-119(ed3) III*	Abby Dernburg (CGC)
CER510	*rpb-2(cer135[rpb-2::GFP$^{\Delta piRNA}$::AID::3xFLAG]) III*	Julián Cerón
CER556	*hcf-1(cer159[hcf-1::GFP$^{\Delta piRNA}$::AID::3xFLAG]) IV*	Julián Cerón
CFJ94	*unc-119(ed3) III; kstSi37 [Cbr-unc-119(kst13)] IV*	Christian Frøkjær-Jensen (CGC)
EG6699	*ttTi5605 II; unc-119(ed3) III; oxEx1578*	Christian Frøkjær-Jensen (CGC)
HAL227	*unc-119(ed3) III; emcSi70 [unc-119p::TIR1::mRuby] IV*	Hannes Lans (CGC)
JA1880	*weSi174[eif-3.Bp::TIR1::linker::mCherry$^{\Delta piRNA}$::tbb-2 3'UTR; unc-119(+)] II; unc-119(ed3) III*	Rhys McDonough and Julie Ahringer
JDW221	*wrdSi50 [mex-5p::TIR1::F2A::mTagBFP2::AID*::NLS::tbb-2 3'UTR] I*	Jordan Ward (CGC)
N2	Wild type (Bristol N2)	CGC
QZ0	Wild type (Bristol N2)	Joy Alcedo

Supplementary Table 5: List of oligos and constructs

Primers

Gene	Forward primer (5'–3')	Reverse primer (5'–3')
daf-2 C-terminus	TTTCGGTGAAAATGAGCATCTA	ACGGGAAGTTTTTGATGGTTTT
TIR1 External (Chr II)	TGGAAATGCTCGGAAGGACT	TGATGTCCGATTGCAGCTTG
TIR1 External (Chr IV)	GGTCCCCATTTCACCAGAGA	GTGGAGGGGCAGTACAGAAT
TIR1[F79]-specific	TCAAGGGAAAGCCACACTTC	N/A
TIR1[F79G]-specific	TCAAGGGAAAGCCACATGGA	N/A
TIR1[F79A]-specific	TCAAGGGAAAGCCACATGCT	N/A
TIR1 Internal	N/A	GAAGTGGGAGAGCCAGTGTC
eif-3.Bp genotyping primers	CGCGGCCTAGGATTCTCTTC (on *eif-3.Bp*)	GAGGAGAGGACGAGGACCTT (on TIR1)
eft-3 promoter	CAACTTCCATTGGTTCTTCCATTGTTTCTG	GGCTGCTACGGAGTGAGCAA
eft-3 3'UTR swap	AGGTAGCTGTAGCGCGATAT	TAAGACATTGCCGCACAGATT
rpb-2 C-terminus	GTAAGCTGCTCTTCCAGGAGT	TTAACCGGAAAAGTCCGTGAT
hcf-1 C-terminus	ATATGGCCCGGCTACTCAAG	GCGGCAAAGTTGGAAAAGGT

crRNAs

Description	Sequence (5'–3', without PAM)
AtTIR1[F79] to AtTIR1[F79A/G]	AGGTTGAAGTCGGCGAAGTG
daf-2 C-terminus	TTTTGGGGGTTTCAGACAAG
nTIR1 *tbb-2* to *eft-3* 3'UTR swap N-terminus	AACTTGTGTCCCAGTTTCGA
nTIR1 *tbb-2* to *eft-3* 3'UTR swap C-terminus	AAAGTCAGGTCTCTGAGCTC

ssDNA Repair templates

Description	Sequence
AtTIR1[F79] to AtTIR1[F79G]	AAGGTCCGTTCCGTCGAGCTCAAGGGAAAGCCACATGGAGCCGACTTCAACCTCGTCCCAGAC GGATGGGGAGG
AtTIR1[F79] to AtTIR1[F79A]	AAGGTCCGTTCCGTCGAGCTCAAGGGAAAGCCACATGCTGCCGACTTCAACCTCGTCCCAGAC GGATGGGGAGG
nTIR1 *tbb-2* to *eft-3* 3'UTR swap	GTAGCTGTAGCGCGATATTGCGATTTGCCATCAAAGCTTGGACATAAACTTAATTAAATCTTCATTG TTGAGTTTATCTTGTTGATTTTTGAATAAATTATCAACTCTTTACTTTTTAATGGGTTATGAAATAAATAA ACATTGAAAACTGATAAACAACGTTCATCTCTCTCAGGAAACGGAGAATCTGTGCGGCAATG

Constructs

Name	Description	Source
**pSEM246**	mosTI(*unc-119*) MCS cloning vector (AmpR)	Christian Frøkjær-Jensen (Addgene plasmid # 159821)
**pNES0036**	MosTI nTIR1 *unc-119* targeting vector (pSEM246 backbone)	This study

Supplementary Table 6: AID and nTIR1 sequences

>1xAID nucleotide sequence

GGATCCGGAGGAGGAGGACCAAAGGACCCAGCCAAGCCACCAGCCAAGGCCCAAGTCGTCGGATGGCCACCAGTCCGTTCCTACCGTA
AGAACGTCATGGTCTCCTGCCAAAAGTCCTCCGGAGGACCAGAGGCCGCCGCCTTCGTCAAGGAGAACCTCTACTTCCAATCCGGAAAGG
ACTACAAGGACCACGACGGAGACTACAAGGACCACGACATCGACTACAAGGACGACGACGACAAG

>1xAID amino acid sequence

GSGGGGPKDPAKPPAKAQVVGWPPVRSYRKNVMVSCQKSSGGPEAAAFVKENLYFQSGKDYKDHDGDYKDHDIDYKDDDDK

>3xAID nucleotide sequence

GGATCCGGAGGAGGAGGACCAAAGGACCCAGCCAAGCCACCAGCCAAGGCCCAAGTCGTCGGATGGCCACCAGTCCGTTCCTACCGTA
AGAACGTCATGGTCTCCTGCCAAAAGTCCTCCGGAGGACCAGAGGCCGCCGCCTTCGTCAAGGGAGCTGGAGCCGGAGCTAAGGAGCCA
AAGGATCCAGCTAAGCCACCAGCTAAGGCTCAAGTTGTTGGCTGGCCACCAGTTCGCTCTTACCGCAAGAACGTTATGGTTTCTTGCCAAAAG
TCTTCTGGTGGTCCAGAAGCTGCTGCTTTCGTTAAGGGAGCTGGAGCAGGAGCCGGAGCTCCAAAAGATCCAGCAAAACCACCAGCAAAAG
CCCAGGTTGTGGGTTGGCCACCAGTGCGTTCATATCGTAAGAACGTGATGGTGTCATGTCAGAAATCATCAGGTGGTCCAGAAGCCGCAGCC
TTCGTGAAAGAGAACCTCTACTTCCAATCCGGAAAGGACTACAAGGACCACGACGGAGATTACAAGGATCACGATATCGATTACAAGGACGAC
GACGACAAG

>3xAID amino acid sequence

GSGGGGPKDPAKPPAKAQVVGWPPVRSYRKNVMVSCQKSSGGPEAAAFVKGAGAGAKEPKDPAKPPAKAQVVGWPPVRSYRKNVMVSCQKS
SGGPEAAAFVKGAGAGAGAPKDPAKPPAKAQVVGWPPVRSYRKNVMVSCQKSSGGPEAAAFVKENLYFQSGKDYKDHDGDYKDHDIDYKDDD
DK

Legend:

AID tag
Linker
TEV site
3x FLAG

>nTIR1(*eft-3p*::TIR1::SL2::NLS::mTagBFP2::*eft-3* 3'UTR) nucleotide sequence

caacttccattggttcttccattgtttctgttaaattaatgaatttttcataaaataaagacattatacaatataaaaatgaagaatttattgaaaataaactgccagagagaaaaagtatgca
acactcccgccgagagtgtttgaaatggtgtacggtacattttcgtgctaggagttagatgtgcaggcagcaacgagagggggagagatttttttgggccttgtgaaattaacgtgagttttct
ggtcatctgactaatcatgttggtttttgttggtttattttgtttttatctttgtttttatccagattaggaaatttaaattttatgaatttataatgaggtcaaacattcagtcccagcgtttttcctgttc
tcactgtttagtcgaatttttattttaggctttcaacaaatgttctaactgtcttatttgtgacctcactttttatatttttttaatttttaaaaatattagaagtttctaggataatttttcgacttttattc
tctctaccgtccgcactcttcttacttttaaattaaattgtttttttttcagttgggaaacactttgctcactccgtagcagccATGCAAAAGCGTATAGCACTTTCATTCCCTGAA
GAGGTCCTGGAACATGTATTTTCATTCATACAGCTTGATAAGgtacgactacctgcctgcctaccgcctAAATTTTTTgtgaagTTTcttcAAAAAAtccagAAAAA
AAAAcaaTTTTcatacgaTTTTTTcccttAAAAttgtgaaTTTTcatgcTTTTTagccccAAAAgtcattaTTTgagAAAAAATTTcatacAAAAAAgTTTTgagAAAtacac
aaTTTTTTAAAtgtaaTTTTcAAATTTTcaaTTTTcaactagAAAAttcacAAAActtgtAAATTTTggaccAAAAcaTTTatacaattacTTTTTTtgaatctaataactacaat
aactacAAATTTTgttcagGATCGAAATTCAGTTTCCTTGGTTTGCAAATCATGGTATGAGATTGAGCGTTGGTGTCGTAGAAAGGTCTTTATCGGTAATT
GTTATGCTGTGTCACCAGCAACCGTAATCAGAAGATTTCCAAAGGTCAGATCAGTCGAGCTCAAGGGAAAGCCGCACTTTGCCGACTTTAACTT
GGTCCCTGATGGCTGGGGCGGATATGTATATCCATGGATCGAAGCAATGTCGTCCTCTTACACCTGGCTGGAAGAGATTAGATTGAAACGTATG
GTTGTCACGGACGATTGCTTAGAGTTGATAGCCAAGTCTTTTAAGAATTTCAAGGTTCTTGTATTGTCCAGTTGCGAAGGATTTTCGACGGACGGT
CTTGCCGCTATTGCCGCCACATGCCGTAATCTTAAGGAATTAGACTTGCGAGAGTCCGACGTGGACGAAGTAAGTGGACACTGGTTATCCCAT
TTCCCAGACACCTACACCTCGCTCGTCTCGCTCAATATATCTTGTCTCGCGAGTGAGGTGTCCTTCTCAGCTCTCGAAAGACTCGTGACGAGAT
GCCCTAACCTCAAGTCTTTGAAGCTGAACCGAGCAGTGCCTCTTGAAAAATTGGCGACCCTCTTGCAGCGTGCACCTCAGCTTGAAGAATTAG
GAACCGGTGGCTACACAGCGGAGGTTCGACCTGACGTGTATAGTGGCCTTTCTGTTGCGCTTAGTGGATGCAAAGAACTGAGATGCCTCTCTG
GTTTCTGGGATGCCGTACCAGCATATCTGCCGGCCGTTTACTCTGTTTGCAGTAGACTGACCACACTGAACCTCTCTTACGCAACCGTGCAGT
CATACGATTTGGTCAAACTGTTATGCCAATGCCCAAAACTCCAGAGACTTTGGGTTTTAGATTACATAGAGgtaagatatgggAAAgaaggAAAAAAccg
agaTTTTacttgAAAAAttgaaTTTTTcgcgggaTTTTcaccAAAAAttgttgaatattcattaTTTcacgctgtAAAAcTTTTAAAAAAAAaatcAAAAActacgttgAAAtcg
cgTTTTTaagcgaaTTTTcttcagaattgccagaTTTTaaccccAAATTTTgcagTTTTTAAAtAAAATTTcaccTTTTcggctcAAAttgtagaTTTTcctgAAAATTTagtac
AAAAAAcaaTTTcctcgtAAATTTTTTcAAATTTTcagGATGCCGGATTAGAAGTTCTGGCGAGTACGTGCAAAGACCTTCGAGAGCTTCGTGTTTTTCCT
TCCGAACCATTCGTTATGGAACCGAATGTGGCACTGACCGAGCAAGGACTGGTCTCCGTTTCCATGGGATGCCCTAAATTAGAGTCGGTACTG
TATTTCTGCCGACAAATGACTAATGCAGCCCTCATTACCATTGCAAGAAATCGACCGAATATGACACGATTCCGATTGTGTATCATCGAGCCGAA
GGCCCCGGATTACCTCACTCTGGAACCACTCGACATCGGTTTCGGTGCGATCGTGGAGCATTGTAAGGATCTTCGTAGACTTTCATTATCCGGT
TTGTTGACGGACAAAGTTTTCGAGTACATAGGAACATACGCCAAAAAAATGGAGATGCTTTCTGTGGCTTTTGCGGGAGACAGTGATTTGGGCTT
GCATCACGTGTTGAGTGGCTGTGACAGTCTGCGTAAACTGGAGATACGAGATTGCCCATTCGGTGACAAAGCTCTTCTTGCGAACGCTTCAAA

ACTCGAGACCATGCGTTCCCTCTGGATGTCTTCGTGCAGTGTGTCCTTTGGAGCATGTAAGCTCCTTGGTCAGAAGATGCCAAAACTCAACGTG
GAGGTCATTGATGAACGTGGCGCTCCAGACTCACGTCCTGAGTCATGCCCGGTTGAAAGAGTCTTTATCTACAGAACCGTTGCGGGACCAAG
ATTCGATATGCCTGGCTTCGTTTGGAATATGGACCAGGATTCTACTATGCGTTTTTCACGTCAGATCATCACGACGAATGGTCTCTAAgctgtctcatc
ctactttcacctagttaactgcttgtcttaaaatctatgcttctctttagtatctaaaattttcctagaagcttacaagtatataaatggtctcttctcaataaaggttgtatatttattcatcttattg
aatctgccatttcctcgtttttgcgagtttatataccttccaattttctttctattgtattttcaacttctaattttaattcagggaaactgcttcaacgcatcATGCCAGCTGCCAAGAGA
GTCAAACTTGACATGGTATCCAAAGGAGAGGAACTGATAAAAGAGAATATGCACATGAAGTTATATATGGAAGgtaagTTTatctAAAAAgTTTTTcattc
AAAAtgtgtAAAAAttcaTTTAAAAtaacccAAAAAtcattaatcctcgataTTTTcagGAACGGTAGATAATCACCATTTCAAATGTACCTCTGAGGGTGAAGG
AAAGCCATATGAAGGCACTCAGACGATGAGAATCAAGGTCGTAGAAGGTGGACCTCTGCCATTCGCCTTCGATATACTTGCCACCTCCTTCTTG
TATGGTTCAAAAACCTTTATAAACCACACGCAGGGTATTCCTGATTTTTTCAAGCAGTCTTTTCCTGAGGGATTCACATGGGAACGAGTAACTACA
TACGAGGACGGAGGTGTCCTGACAGCAACACAAGATACTTCATTACAAGATGGTTGTCTTATATATAATGTGAAGATAAGAGgtaTTTTcctgcaTTTTT
caactgggAAAAtgAAAgAAAAtcgataaTTTcagGTGTCAATTTCACGAGTAACGGCCCGGTGATGCAGAAAAAGACGTTAGGCTGGGAGGCGTTCA
CAGAGACGCTTTATCCAGCTGATGGTGGACTCGAGGGCAGAAATGATATGGCTCTCAAGTTGGTTGGAGGATCGCATCTTATTGCAAATGCCAA
AACAACATATAGATCTAAAAAACCGGCCAAAAACCTTAAGATGCCTGGCGTCTACTACGTGGATTATCGTCTCGAACGAATCAAAGAAGCTAATA
ACGAGACTTATGTCGAGCAACACGAGGTAGCTGTAGCGCGATATTGCGATTTGCCATCAAAGCTTGGACATAAACTTAATTAAatcttcattgttgagttt
atcttgttgatttttgaataaattatcaactctttactttttaatgggttatgaaataaataaacattgaaaactgataaacaacgttcatctct

Legend:

eft-3 promoter
nTIR1
SL2 (gpd-2/3 intergenic sequence)
c-Myc Nuclear Localization Signal (NLS)
mTagBFP2
eft-3 3'UTR

>nTIR1(*eft-3p*::TIR1::SL2::NLS::mTagBFP2::*eft-3* 3'UTR) amino acid sequence

MQKRIALSFPEEVLEHVFSFIQLDKDRNSVSLVCKSWYEIERWCRRKVFIGNCYAVSPATVIRRFPKVRSVELKGKPHFADFNLVPDGWGGYVYP
WIEAMSSSYTWLEEIRLKRMVVTDDCLELIAKSFKNFKVLVLSSCEGFSTDGLAAIAATCRNLKELDLRESDVDEVSGHWLSHFPDTYTSLVSLNIS
CLASEVSFSALERLVTRCPNLKSLKLNRAVPLEKLATLLQRAPQLEELGTGGYTAEVRPDVYSGLSVALSGCKELRCLSGFWDAVPAYLPAVYSVC
SRLTTLNLSYATVQSYDLVKLLCQCPKLQRLWVLDYIEDAGLEVLASTCKDLRELRVFPSEPFVMEPNVALTEQGLVSVSMGCPKLESVLYFCRQM
TNAALITIARNRPNMTRFRLCIIEPKAPDYLTLEPLDIGFGAIVEHCKDLRRLSLSGLLTDKVFEYIGTYAKKMEMLSVAFAGDSDLGLHHVLSGCDSL
RKLEIRDCPFGDKALLANASKLETMRSLWMSSCSVSFGACKLLGQKMPKLNVEVIDERGAPDSRPESCPVERVFIYRTVAGPRFDMPGFVWNM
DQDSTMRFSRQIITTNGL*MPAAKRVKLDMVSKGEELIKENMHMKLYMEGTVDNHHFKCTSEGEGKPYEGTQTMRIKVVEGGPLPFAFDILATSFL
YGSKTFINHTQGIPDFFKQSFPEGFWERVTTYEDGGVLTATQDTSLQDGCLIYNVKIRGVNFTSNGPVMQKKTLGWEAFTETLYPADGGLEGRND
MALKLVGGSHLIANAKTTYRSKKPAKNLKMPGVYYVDYRLERIKEANNETYVEQHEVAVARYCDLPSKLGHKLN*

Legend:

nTIR1
c-Myc Nuclear Localization Signal (NLS)
mTagBFP2

Supplementary Table 7: List of sample sizes for each experimental condition

Figure	TIR1 variant	Compound	Concentration (μM)	N	Units	Promoter	Degron
Fig. 1 d, Fig. S2 a-b	none	none	0	132	individuals	NA	NA
Fig. 1 d, Fig. S2 a	none	K-NAA	750	82	individuals	NA	NA
Fig. 1 d, Fig. S2 a	none	5-Ad-IAA	1.875	115	individuals	NA	NA
Fig. 1 d, Fig. S2 a	none	5-Ph-IAA	1.875	101	individuals	NA	NA
Fig. 1 c-d, Fig. 2 d	F79	K-NAA	0	96	individuals	*eft-3p*	1x
Fig. 1 c-d, Fig. 2 d	F79	K-NAA	10	100	individuals	*eft-3p*	1x
Fig. 1 c-d, Fig. 2 d	F79	K-NAA	250	87	individuals	*eft-3p*	1x
Fig. 1 c-d, Fig. 2 d	F79	K-NAA	500	103	individuals	*eft-3p*	1x
Fig. 1 c-d, Fig. 2 d	F79	K-NAA	750	117	individuals	*eft-3p*	1x
Fig. 1 c-d, Fig. 2 e, Fig. S3 b	F79A	none	0	135	individuals	*eft-3p*	1x
Fig. 1 c-d, Fig. 2 e	F79A	5-Ad-IAA	0.025	150	individuals	*eft-3p*	1x
Fig. 1 c-d, Fig. 2 e	F79A	5-Ad-IAA	0.625	105	individuals	*eft-3p*	1x
Fig. 1 c-d, Fig. 2 e	F79A	5-Ad-IAA	1.25	111	individuals	*eft-3p*	1x
Fig. 1 c-d, Fig. 2 e	F79A	5-Ad-IAA	1.875	116	individuals	*eft-3p*	1x
Fig. 1 c-d, Fig. 2 f, Fig. S3 b	F79G	none	0	108	individuals	*eft-3p*	1x
Fig. 1 c-d, Fig. 2 f	F79G	5-Ph-IAA	0.025	101	individuals	*eft-3p*	1x
Fig. 1 c-d, Fig. 2 f	F79G	5-Ph-IAA	0.625	122	individuals	*eft-3p*	1x
Fig. 1 c-d, Fig. 2 f	F79G	5-Ph-IAA	1.25	119	individuals	*eft-3p*	1x
Fig. 1 c-d, Fig. 2 f	F79G	5-Ph-IAA	1.875	144	individuals	*eft-3p*	1x
Fig. 1 e	F79A	none	0	9	populations	*eft-3p*	1x
Fig. 1 e	F79G	none	0	9	populations	*eft-3p*	1x
Fig. 1 e	F79	none	0	6	populations	*eft-3p*	1x
Fig. 1 e	F79A	5-Ad-IAA	0.001	9	populations	*eft-3p*	1x
Fig. 1 e	F79G	5-Ph-IAA	0.001	9	populations	*eft-3p*	1x
Fig. 1 e	F79A	5-Ad-IAA	0.005	9	populations	*eft-3p*	1x
Fig. 1 e	F79G	5-Ph-IAA	0.005	9	populations	*eft-3p*	1x
Fig. 1 e	F79A	5-Ad-IAA	0.01	9	populations	*eft-3p*	1x
Fig. 1 e	F79G	5-Ph-IAA	0.01	9	populations	*eft-3p*	1x
Fig. 1 e	F79A	5-Ad-IAA	0.025	9	populations	*eft-3p*	1x
Fig. 1 e	F79G	5-Ph-IAA	0.025	9	populations	*eft-3p*	1x
Fig. 1 e	F79A	5-Ad-IAA	0.05	9	populations	*eft-3p*	1x
Fig. 1 e	F79G	5-Ph-IAA	0.05	9	populations	*eft-3p*	1x
Fig. 1 e	F79A	5-Ad-IAA	0.1	9	populations	*eft-3p*	1x
Fig. 1 e	F79G	5-Ph-IAA	0.1	9	populations	*eft-3p*	1x
Fig. 1 e	F79	K-NAA	0.1	9	populations	*eft-3p*	1x
Fig. 1 e	F79	K-NAA	1	9	populations	*eft-3p*	1x
Fig. 1 e	F79	K-NAA	10	9	populations	*eft-3p*	1x
Fig. 1 f	F79	none	0	3	plates	*eft-3p*	1x
Fig. 1 f	F79A	none	0	3	plates	*eft-3p*	1x
Fig. 1 f	F79G	none	0	3	plates	*eft-3p*	1x

Fig. 1 f	F79	none	0	3	plates	*eft-3p*	1x
Fig. 1 f	F79A	none	0	3	plates	*eft-3p*	1x
Fig. 1 f	F79G	none	0	3	plates	*eft-3p*	1x
Fig. 1 f	F79	none	0	3	plates	*eft-3p*	1x
Fig. 1 f	F79A	none	0	3	plates	*eft-3p*	1x
Fig. 1 f	F79G	none	0	3	plates	*eft-3p*	1x
Fig. 1 f	F79	5-Ad-IAA	0.001	3	plates	*eft-3p*	1x
Fig. 1 f	F79A	5-Ad-IAA	0.001	3	plates	*eft-3p*	1x
Fig. 1 f	F79G	5-Ad-IAA	0.001	3	plates	*eft-3p*	1x
Fig. 1 f	F79	5-Ph-IAA	0.001	3	plates	*eft-3p*	1x
Fig. 1 f	F79A	5-Ph-IAA	0.001	3	plates	*eft-3p*	1x
Fig. 1 f	F79G	5-Ph-IAA	0.001	3	plates	*eft-3p*	1x
Fig. 1 f	F79	5-Ad-IAA	0.005	3	plates	*eft-3p*	1x
Fig. 1 f	F79A	5-Ad-IAA	0.005	3	plates	*eft-3p*	1x
Fig. 1 f	F79G	5-Ad-IAA	0.005	3	plates	*eft-3p*	1x
Fig. 1 f	F79	5-Ph-IAA	0.005	3	plates	*eft-3p*	1x
Fig. 1 f	F79A	5-Ph-IAA	0.005	3	plates	*eft-3p*	1x
Fig. 1 f	F79G	5-Ph-IAA	0.005	3	plates	*eft-3p*	1x
Fig. 1 f	F79	5-Ad-IAA	0.01	3	plates	*eft-3p*	1x
Fig. 1 f	F79A	5-Ad-IAA	0.01	3	plates	*eft-3p*	1x
Fig. 1 f	F79G	5-Ad-IAA	0.01	3	plates	*eft-3p*	1x
Fig. 1 f	F79	5-Ph-IAA	0.01	3	plates	*eft-3p*	1x
Fig. 1 f	F79A	5-Ph-IAA	0.01	3	plates	*eft-3p*	1x
Fig. 1 f	F79G	5-Ph-IAA	0.01	3	plates	*eft-3p*	1x
Fig. 1 f	F79	5-Ad-IAA	0.025	3	plates	*eft-3p*	1x
Fig. 1 f	F79A	5-Ad-IAA	0.025	3	plates	*eft-3p*	1x
Fig. 1 f	F79G	5-Ad-IAA	0.025	3	plates	*eft-3p*	1x
Fig. 1 f	F79	5-Ph-IAA	0.025	3	plates	*eft-3p*	1x
Fig. 1 f	F79A	5-Ph-IAA	0.025	3	plates	*eft-3p*	1x
Fig. 1 f	F79G	5-Ph-IAA	0.025	3	plates	*eft-3p*	1x
Fig. 1 f	F79	5-Ad-IAA	0.05	3	plates	*eft-3p*	1x
Fig. 1 f	F79A	5-Ad-IAA	0.05	3	plates	*eft-3p*	1x
Fig. 1 f	F79G	5-Ad-IAA	0.05	3	plates	*eft-3p*	1x
Fig. 1 f	F79	5-Ph-IAA	0.05	3	plates	*eft-3p*	1x
Fig. 1 f	F79A	5-Ph-IAA	0.05	3	plates	*eft-3p*	1x
Fig. 1 f	F79G	5-Ph-IAA	0.05	3	plates	*eft-3p*	1x
Fig. 1 f	F79	5-Ad-IAA	0.1	3	plates	*eft-3p*	1x
Fig. 1 f	F79A	5-Ad-IAA	0.1	3	plates	*eft-3p*	1x
Fig. 1 f	F79G	5-Ad-IAA	0.1	3	plates	*eft-3p*	1x
Fig. 1 f	F79	5-Ph-IAA	0.1	3	plates	*eft-3p*	1x
Fig. 1 f	F79A	5-Ph-IAA	0.1	3	plates	*eft-3p*	1x
Fig. 1 f	F79G	5-Ph-IAA	0.1	3	plates	*eft-3p*	1x
Fig. 1 f	F79	K-NAA	0.1	3	plates	*eft-3p*	1x
Fig. 1 f	F79A	K-NAA	0.1	3	plates	*eft-3p*	1x

Fig. 1 f	F79G	K-NAA	0.1	3	plates	*eft-3p*	1x
Fig. 1 f	F79	K-NAA	1	3	plates	*eft-3p*	1x
Fig. 1 f	F79A	K-NAA	1	3	plates	*eft-3p*	1x
Fig. 1 f	F79G	K-NAA	1	3	plates	*eft-3p*	1x
Fig. 1 f	F79	K-NAA	10	3	plates	*eft-3p*	1x
Fig. 1 f	F79A	K-NAA	10	3	plates	*eft-3p*	1x
Fig. 1 f	F79G	K-NAA	10	3	plates	*eft-3p*	1x
Fig. 1 f	F79	K-NAA	25	3	plates	*eft-3p*	1x
Fig. 1 f	F79A	K-NAA	25	3	plates	*eft-3p*	1x
Fig. 1 f	F79G	K-NAA	25	3	plates	*eft-3p*	1x
Fig. 1 f	F79	K-NAA	50	3	plates	*eft-3p*	1x
Fig. 1 f	F79A	K-NAA	50	3	plates	*eft-3p*	1x
Fig. 1 f	F79G	K-NAA	50	3	plates	*eft-3p*	1x
Fig. 1 f	F79	K-NAA	100	3	plates	*eft-3p*	1x
Fig. 1 f	F79A	K-NAA	100	3	plates	*eft-3p*	1x
Fig. 1 f	F79G	K-NAA	100	3	plates	*eft-3p*	1x
Fig. 1 f	F79	K-NAA	250	3	plates	*eft-3p*	1x
Fig. 1 f	F79A	K-NAA	250	3	plates	*eft-3p*	1x
Fig. 1 f	F79G	K-NAA	250	3	plates	*eft-3p*	1x
Fig. 1 f	F79	K-NAA	500	3	plates	*eft-3p*	1x
Fig. 1 f	F79A	K-NAA	500	3	plates	*eft-3p*	1x
Fig. 1 f	F79G	K-NAA	500	3	plates	*eft-3p*	1x
Fig. 2 d	F79	none	0	172	individuals	*eft-3p*	1x
Fig. 2 d	F79	none	0	85	individuals	*eft-3p*	1x
Fig. 2 d	F79	K-NAA	10	193	individuals	*eft-3p*	1x
Fig. 2 d	F79	K-NAA	250	206	individuals	*eft-3p*	1x
Fig. 2 d	F79	K-NAA	500	194	individuals	*eft-3p*	1x
Fig. 2 d	F79	K-NAA	750	349	individuals	*eft-3p*	1x
Fig. 2 d	F79	none	0	225	individuals	*eft-3p*	3x
Fig. 2 d	F79	none	0	81	individuals	*eft-3p*	3x
Fig. 2 d	F79	K-NAA	10	222	individuals	*eft-3p*	3x
Fig. 2 d	F79	K-NAA	250	252	individuals	*eft-3p*	3x
Fig. 2 d	F79	K-NAA	500	198	individuals	*eft-3p*	3x
Fig. 2 d	F79	K-NAA	750	344	individuals	*eft-3p*	3x
Fig. 2 d	F79	none	0	141	individuals	*eif-3.Bp*	3x
Fig. 2 d	F79	none	0	83	individuals	*eif-3.Bp*	3x
Fig. 2 d	F79	K-NAA	10	127	individuals	*eif-3.Bp*	3x
Fig. 2 d	F79	K-NAA	250	117	individuals	*eif-3.Bp*	3x
Fig. 2 d	F79	K-NAA	500	138	individuals	*eif-3.Bp*	3x
Fig. 2 d	F79	K-NAA	750	110	individuals	*eif-3.Bp*	3x
Fig. 2 d	F79	none	0	94	individuals	*eif-3.Bp*	1x
Fig. 2 d	F79	none	0	82	individuals	*eif-3.Bp*	1x
Fig. 2 d	F79	K-NAA	10	120	individuals	*eif-3.Bp*	1x
Fig. 2 d	F79	K-NAA	250	111	individuals	*eif-3.Bp*	1x

Fig. 2 d	F79	K-NAA	500	173	individuals	*eif-3.Bp*	1x
Fig. 2 d	F79	K-NAA	750	137	individuals	*eif-3.Bp*	1x
Fig. 2 e	F79A	none	0	109	individuals	*eft-3p*	1x
Fig. 2 e	F79A	none	0	138	individuals	*eft-3p*	3x
Fig. 2 e	F79A	none	0	127	individuals	*eft-3p*	3x
Fig. 2 e	F79A	5-Ad-IAA	0.025	146	individuals	*eft-3p*	3x
Fig. 2 e	F79A	5-Ad-IAA	0.625	112	individuals	*eft-3p*	3x
Fig. 2 e	F79A	5-Ad-IAA	1.25	115	individuals	*eft-3p*	3x
Fig. 2 e	F79A	5-Ad-IAA	1.875	117	individuals	*eft-3p*	3x
Fig. 2 f	F79G	none	0	121	individuals	*eft-3p*	1x
Fig. 2 f	F79G	none	0	155	individuals	*eft-3p*	3x
Fig. 2 f	F79G	none	0	88	individuals	*eft-3p*	3x
Fig. 2 f	F79G	5-Ph-IAA	0.025	130	individuals	*eft-3p*	3x
Fig. 2 f	F79G	5-Ph-IAA	0.625	128	individuals	*eft-3p*	3x
Fig. 2 f	F79G	5-Ph-IAA	1.25	111	individuals	*eft-3p*	3x
Fig. 2 f	F79G	5-Ph-IAA	1.875	108	individuals	*eft-3p*	3x
Fig. 2 g	F79	none	0	6	plates	*eft-3p*	1x
Fig. 2 g	F79	none	0	9	plates	*eif-3.Bp*	1x
Fig. 2 g	F79	none	0	6	plates	*eft-3p*	3x
Fig. 2 g	F79	none	0	12	plates	*eif-3.Bp*	3x
Fig. 2 g	F79	K-NAA	0.1	9	plates	*eft-3p*	1x
Fig. 2 g	F79	K-NAA	0.1	9	plates	*eft-3p*	3x
Fig. 2 g	F79	K-NAA	1	9	plates	*eft-3p*	1x
Fig. 2 g	F79	K-NAA	1	9	plates	*eft-3p*	3x
Fig. 2 g	F79	K-NAA	10	9	plates	*eft-3p*	1x
Fig. 2 g	F79	K-NAA	10	9	plates	*eif-3.Bp*	1x
Fig. 2 g	F79	K-NAA	10	9	plates	*eft-3p*	3x
Fig. 2 g	F79	K-NAA	10	12	plates	*eif-3.Bp*	3x
Fig. 2 g	F79	K-NAA	25	6	plates	*eif-3.Bp*	1x
Fig. 2 g	F79	K-NAA	25	12	plates	*eif-3.Bp*	3x
Fig. 2 g	F79	K-NAA	50	6	plates	*eif-3.Bp*	1x
Fig. 2 g	F79	K-NAA	50	12	plates	*eif-3.Bp*	3x
Fig. 2 g	F79	K-NAA	100	6	plates	*eif-3.Bp*	1x
Fig. 2 g	F79	K-NAA	100	15	plates	*eif-3.Bp*	3x
Fig. 2 g	F79	K-NAA	250	6	plates	*eif-3.Bp*	1x
Fig. 2 g	F79	K-NAA	250	9	plates	*eif-3.Bp*	3x
Fig. 2 g	F79	K-NAA	500	9	plates	*eif-3.Bp*	1x
Fig. 2 g	F79	K-NAA	500	9	plates	*eif-3.Bp*	3x
Fig. 2 h	F79A	none	0	9	plates	*eft-3p*	1x
Fig. 2 h	F79A	none	0	9	plates	*eft-3p*	3x
Fig. 2 h	F79A	5-Ad-IAA	0.001	9	plates	*eft-3p*	1x
Fig. 2 h	F79A	5-Ad-IAA	0.001	9	plates	*eft-3p*	3x
Fig. 2 h	F79A	5-Ad-IAA	0.005	9	plates	*eft-3p*	1x
Fig. 2 h	F79A	5-Ad-IAA	0.005	9	plates	*eft-3p*	3x

Fig. 2 h	F79A	5-Ad-IAA	0.01	9	plates	*eft-3p*	1x
Fig. 2 h	F79A	5-Ad-IAA	0.01	9	plates	*eft-3p*	3x
Fig. 2 h	F79A	5-Ad-IAA	0.025	9	plates	*eft-3p*	1x
Fig. 2 h	F79A	5-Ad-IAA	0.025	9	plates	*eft-3p*	3x
Fig. 2 h	F79A	5-Ad-IAA	0.05	9	plates	*eft-3p*	1x
Fig. 2 h	F79A	5-Ad-IAA	0.05	9	plates	*eft-3p*	3x
Fig. 2 h	F79A	5-Ad-IAA	0.1	9	plates	*eft-3p*	1x
Fig. 2 h	F79A	5-Ad-IAA	0.1	9	plates	*eft-3p*	3x
Fig. 2 i	F79G	none	0	9	plates	*eft-3p*	1x
Fig. 2 i	F79G	none	0	9	plates	*eft-3p*	3x
Fig. 2 i	F79G	5-Ph-IAA	0.001	9	plates	*eft-3p*	1x
Fig. 2 i	F79G	5-Ph-IAA	0.001	9	plates	*eft-3p*	3x
Fig. 2 i	F79G	5-Ph-IAA	0.005	9	plates	*eft-3p*	1x
Fig. 2 i	F79G	5-Ph-IAA	0.005	9	plates	*eft-3p*	3x
Fig. 2 i	F79G	5-Ph-IAA	0.01	9	plates	*eft-3p*	1x
Fig. 2 i	F79G	5-Ph-IAA	0.01	9	plates	*eft-3p*	3x
Fig. 2 i	F79G	5-Ph-IAA	0.025	9	plates	*eft-3p*	1x
Fig. 2 i	F79G	5-Ph-IAA	0.025	9	plates	*eft-3p*	3x
Fig. 2 i	F79G	5-Ph-IAA	0.05	9	plates	*eft-3p*	1x
Fig. 2 i	F79G	5-Ph-IAA	0.05	9	plates	*eft-3p*	3x
Fig. 2 i	F79G	5-Ph-IAA	0.1	9	plates	*eft-3p*	1x
Fig. 2 i	F79G	5-Ph-IAA	0.1	9	plates	*eft-3p*	3x
Fig. 5 c,e	F79/F79G	none	0	10	individuals	*ges-119p/unc-119p*	1x
Fig. 5 c	F79/F79G	K-NAA	50	10	individuals	*ges-119p/unc-119p*	1x
Fig. 5 c	F79/F79G	K-NAA	100	10	individuals	*ges-119p/unc-119p*	1x
Fig. 5 c	F79/F79G	K-NAA	250	10	individuals	*ges-119p/unc-119p*	1x
Fig. 5 c,e	F79/F79G	K-NAA	500	10	individuals	*ges-119p/unc-119p*	1x
Fig. 5 d	F79/F79G	5-Ph-IAA	0.05	10	individuals	*ges-119p/unc-119p*	1x
Fig. 5 d	F79/F79G	5-Ph-IAA	0.1	10	individuals	*ges-119p/unc-119p*	1x
Fig. 5 d	F79/F79G	5-Ph-IAA	0.25	10	individuals	*ges-119p/unc-119p*	1x
Fig. 5 d	F79/F79G	5-Ph-IAA	0.5	10	individuals	*ges-119p/unc-119p*	1x
Fig. 5 d-e	F79/F79G	5-Ph-IAA	1	10	individuals	*ges-119p/unc-119p*	1x
Fig. 5 f	F79/F79G	Dual Auxin	1 uM 5-Ph-IAA + 500 uM K-NAA	9	individuals	*ges-119p/unc-119p*	1x
Fig. 5 h-i	F79/F79G	None	0	10	individuals	*ges-119p/unc-119p*	1x
Fig. 5 h	F79/F79G	K-NAA	33	10	individuals	*ges-119p/unc-119p*	1x
Fig. 5 h	F79/F79G	K-NAA	67	10	individuals	*ges-119p/unc-119p*	1x
Fig. 5 h	F79/F79G	K-NAA	125	10	individuals	*ges-119p/unc-119p*	1x
Fig. 5 h	F79/F79G	K-NAA	250	10	individuals	*ges-119p/unc-119p*	1x
Fig. 5 i	F79/F79G	5-Ph-IAA	0.025	10	individuals	*ges-119p/unc-119p*	1x
Fig. 5 i	F79/F79G	5-Ph-IAA	0.05	10	individuals	*ges-119p/unc-119p*	1x

Fig. 5 i	F79/F79G	5-Ph-IAA	1.00	10	individuals	*ges-119p/unc-119p*	1x
Fig. 5 i	F79/F79G	5-Ph-IAA	2.00	10	individuals	*ges-119p/unc-119p*	1x
Fig. 5 j	F79/F79G	Dual Auxin	2.0 uM 5-Ph-IAA + 250 uM K-NAA	10	individuals	*ges-119p/unc-119p*	1x
Fig. 6 d-e	unc-54 3'UTR	none	0	5	individuals	*eft-3p*	1x
Fig. 6 d-e	unc-54 3'UTR	K-NAA	200	5	individuals	*eft-3p*	1x
Fig. 6 d-e	eft-3p 3'UTR	none	0	5	individuals	*eft-3p*	1x
Fig. 6 d-e	eft-3p 3'UTR	K-NAA	200	5	individuals	*eft-3p*	1x
Fig. S2 c (Day 0)	F79	none	0	10	individuals	*eft-3p*	None
Fig. S2 c (Day 0)	F79	none	0	10	individuals	*eif-3.Bp*	None
Fig. S3 a (Day 0)	F79	none	0	104	individuals	*eft-3p*	1x
Fig. S3 a (Day 0)	F79	K-NAA	10	300	individuals	*eft-3p*	1x
Fig. S3 a (Day 0)	F79G	none	0	98	individuals	*eft-3p*	1x
Fig. S3 a (Day 0)	F79G	5-Ph-IAA	0.05	313	individuals	*eft-3p*	1x
Fig. S3 a (Day 0)	F79A	none	0	112	individuals	*eft-3p*	1x
Fig. S3 a (Day 0)	F79A	5-Ad-IAA	0.025	294	individuals	*eft-3p*	1x
Fig. S3 a (Day 7)	F79	none	0	111	individuals	*eft-3p*	1x
Fig. S3 a (Day 7)	F79	K-NAA	10	307	individuals	*eft-3p*	1x
Fig. S3 a (Day 7)	F79G	none	0	107	individuals	*eft-3p*	1x
Fig. S3 a (Day 7)	F79G	5-Ph-IAA	0.05	303	individuals	*eft-3p*	1x
Fig. S3 a (Day 7)	F79A	none	0	105	individuals	*eft-3p*	1x
Fig. S3 a (Day 7)	F79A	5-Ad-IAA	0.025	297	individuals	*eft-3p*	1x
Fig. S3 a (Day 14)	F79	none	0	135	individuals	*eft-3p*	1x
Fig. S3 a (Day 14)	F79	K-NAA	10	282	individuals	*eft-3p*	1x
Fig. S3 a (Day 14)	F79G	none	0	152	individuals	*eft-3p*	1x
Fig. S3 a (Day 14)	F79G	5-Ph-IAA	0.05	296	individuals	*eft-3p*	1x
Fig. S3 a (Day 14)	F79A	none	0	148	individuals	*eft-3p*	1x
Fig. S3 a (Day 14)	F79A	5-Ad-IAA	0.025	294	individuals	*eft-3p*	1x
Fig. S3 a (Day 21)	F79	none	0	129	individuals	*eft-3p*	1x
Fig. S3 a (Day 21)	F79	K-NAA	10	294	individuals	*eft-3p*	1x
Fig. S3 a (Day 21)	F79G	none	0	138	individuals	*eft-3p*	1x
Fig. S3 a (Day 21)	F79G	5-Ph-IAA	0.05	289	individuals	*eft-3p*	1x
Fig. S3 a (Day 21)	F79A	none	0	137	individuals	*eft-3p*	1x
Fig. S3 a (Day 21)	F79A	5-Ad-IAA	0.025	302	individuals	*eft-3p*	1x
Fig. S3 a (Day 28)	F79	none	0	97	individuals	*eft-3p*	1x
Fig. S3 a-b (Day 28, seeded)	F79	K-NAA	10	299	individuals	*eft-3p*	1x
Fig. S3 a (Day 28)	F79G	none	0	154	individuals	*eft-3p*	1x
Fig. S3 a (Day 28)	F79G	5-Ph-IAA	0.05	311	individuals	*eft-3p*	1x
Fig. S3 a (Day 28)	F79A	none	0	120	individuals	*eft-3p*	1x
Fig. S3 a (Day 28)	F79A	5-Ad-IAA	0.025	291	individuals	*eft-3p*	1x
Fig. S3 b (Day 28, unseeded)	F79	K-NAA	10	290	individuals	*eft-3p*	1x
Fig. S3 c	F79	None	0	7	plates	*eft-3p*	1x
Fig. S3 c	F79	None	0	7	plates	*eft-3p*	3x

Fig. S3 c	F79	None	0	7	plates	*eif-3.Bp*	1x
Fig. S3 c	F79	None	0	7	plates	*eif-3.Bp*	3x
Fig. S3 c	F79A	None	0	7	plates	*eft-3p*	1x
Fig. S3 c	F79A	None	0	7	plates	*eft-3p*	3x
Fig. S3 c	F79G	None	0	7	plates	*eft-3p*	1x
Fig. S3 c	F79G	None	0	7	plates	*eft-3p*	3x
Fig. S6 b-c	None	None	0	5	individuals	*eft-3p*	1x
Fig. S6 b-c	None	K-NAA	200	5	individuals	*eft-3p*	1x
Fig. S6 b-c	F79	None	0	5	individuals	*eft-3p*	1x
Fig. S6 b-c	F79	K-NAA	200	5	individuals	*eft-3p*	1x
Fig. S6 b-c	F79G	None	0	5	individuals	*mex-5p*	1x
Fig. S6 b-c	F79G	K-NAA	200	5	individuals	*mex-5p*	1x
Fig. S6 b-c	F79/F79G	None	0	5	individuals	*eft-3p/mex-5p*	1x
Fig. S6 b-c	F79/F79G	K-NAA	200	5	individuals	*eft-3p/mex-5p*	1x

**Figure**	**TIR1 variant**	**Compound**	**Concentration**	**N**	**Units**	**Promoter**	**Degron**	**RNAi**
Fig. S7 c-d	F79	None	0	5	Individuals	*eft-3p*	1x	EV
Fig. S7 c-d	F79	K-NAA	200	5	Individuals	*eft-3p*	1x	EV
Fig. S7 c-d	F79	None	0	5	Individuals	*eft-3p*	1x	*hrde-1*
Fig. S7 c-d	F79	K-NAA	200	5	Individuals	*eft-3p*	1x	*hrde-1*
