# Supplementary material for: Engineering the auxin-inducible degron system for tunable in vivo control of organismal physiology
Source: Nat Commun. 2025 Dec 12;16:10848. doi: 10.1038/s41467-025-66347-x (PMC12700905; doi:10.1038/s41467-025-66347-x)
Supplement: Supplementary file 3 — Description of Additional Supplementary Files [file 41467_2025_66347_MOESM3_ESM.pdf]

Description of Additional Supplementary Files

File Name: Supplementary Data 1
Description: Full expression data of differentially expressed genes in wild-type QZ0 worms upon exposure to auxin analogs.

File Name: Supplementary Data 2
Description: Full expression data of differentially expressed genes in strains with different transgenes at the TIR1 and daf-2 loci in the absence of KNAA, 5-Ph-IAA, and 5-Ad-IAA.

File Name: Supplementary Data 3
Description: Differentially expressed genes in DAF-2::AID; TIR1- strains upon exposure to their corresponding activating ligands.

File Name: Supplementary Data 4
Description: Gene set enrichment analysis for the "off-target" activities of AID system components, including the activity of compounds on wildtype animals and the activity of TIR1 transgene variants in the absence of any other AID component.
